# Optimization of lipids’ ultrasonic extraction and production from *Chlorella* sp. using response-surface methodology

**DOI:** 10.1186/s12944-018-0702-z

**Published:** 2018-04-17

**Authors:** Bilel Hadrich, Ismahen Akremi, Mouna Dammak, Mohamed Barkallah, Imen Fendri, Slim Abdelkafi

**Affiliations:** 1 Unité de Biotechnologie des Algues, Biological Engineering Department, National School of Engineers of Sfax, University of Sfax, Sfax, Tunisia; 2 Laboratoire de Biotechnologie végétale appliquée à l’amélioration des cultures, Faculty of Sciences of Sfax, University of Sfax, Sfax, Tunisia

**Keywords:** Microalgae, Lipids, Extraction, Biodiesel, Production, Response-surface methodology

## Abstract

**Background:**

Three steps are very important in order to produce microalgal lipids: (1) controlling microalgae cultivation via experimental and modeling investigations, (2) optimizing culture conditions to maximize lipids production and to determine the fatty acid profile the most appropriate for biodiesel synthesis, and (3) optimizing the extraction of the lipids accumulated in the microalgal cells.

**Methods:**

Firstly, three kinetics models, namely logistic, logistic-with-lag and modified Gompertz, were tested to fit the experimental kinetics of the *Chlorella* sp. microalga culture established on standard conditions. Secondly, the response-surface methodology was used for two optimizations in this study. The first optimization was established for lipids production from *Chlorella* sp. culture under different culture conditions. In fact, different levels of nitrate concentrations, salinities and light intensities were applied to the culture medium in order to study their influences on lipids production and determine their fatty acid profile. The second optimization was concerned with the lipids extraction factors: ultrasonic’s time and temperature, and chloroform-methanol solvent ratio.

**Results:**

All models (logistic, logistic-with-lag and modified Gompertz) applied for the experimental kinetics of *Chlorella* sp. show a very interesting fitting quality. The logistic model was chosen to describe the *Chlorella* sp. kinetics, since it yielded the most important statistical criteria: coefficient of determination of the order of 94.36%; adjusted coefficient of determination equal to 93.79% and root mean square error reaching 3.685 cells **·** ml^− 1^.

Nitrate concentration and the two interactions involving the light intensity (Nitrate concentration × light intensity, and salinities × light intensity) showed a very significant influence on lipids production in the first optimization (*p* < 0.05). Yet, only the quadratic term of chloroform-methanol solvent ratio showed a significant influence on lipids extraction relative to the second step of optimization (*p* < 0.05).

The two most abundant fatty acid methyl esters (≈72%) derived from the *Chlorella* sp. microalga cultured in the determined optimal conditions are: palmitic acid (C16:0) and oleic acid (C18:1) with the corresponding yields of 51.69% and 20.55% of total fatty acids, respectively.

**Conclusions:**

Only the nitrate deficiency and the high intensity of light can influence the microalgal lipids production. The corresponding fatty acid methyl esters composition is very suitable for biodiesel production. Lipids extraction is efficient only over long periods of time when using a solvent with a 2/1 chloroform/methanol ratio.

## Background

The biofuel derived from microalgae is considered one of the most promising and important renewable energy sources because of its many advantages. First, microalgae are photosynthetic microorganisms that adapt rapidly to new environments. In addition, compared to other bioenergy sources like soybean, corn, …, microalgae are characterized by high growth rates, high lipid production capacity, high CO_2_ fixation rates, and low cultivation space requirement [1]. The resultant biofuel is environment-friendly and does not exacerbate the carbon footprint.

Three important steps can be considered to produce biofuel from microalgae. First, controlling microalgae cultivation is a step of paramount importance. Actually, growth kinetic models are needed to approve this objective, and, thereafter, culture conditions can be optimized. In the corresponding literature, many models were established in order to study the growth kinetics of many microalgae and to determine the growth kinetics’ characterizations [1–4].

Second, the optimization of culture conditions to maximize lipids productivity is another key step. Many authors are currently working towards the identification of the optimal culture conditions in order to maximize lipids accumulation in microalgal cells. The most studied factors in this vein are: light intensity, salinity, pH, nitrogen limitation, phosphorus limitation, … etc. [1, 3, 5–7].

The third crucial step is extraction optimization of the lipids accumulated in the microalgal cells. It consists in the working on lipids extraction that is in continuous development. Several methods have been used to perform a pretreatment leading to the effective extraction of lipids [8, 9]. Ultrasonic extraction is among the newest lipids extraction technologies. Ultrasonic exposition with the aim of lipids extraction showed the highest performance compared to other techniques [8]. The optimization of the relative conditions can be very useful in the biodiesel field. About the used solvents for lipids extraction, Folch et al. [10] and Park et al. [9] recommend chloroform/methanol as better solvent mixture for lipids extraction from microalgae. In fact, it is more suitable for microalgal lipids compared to other mixtures (e.g. Hexane; Hexane/Methanol).

This work aims to range over three principal subjects: the experimental and modeling studies of the growth kinetics of *Chlorella* sp. microalga, the optimization of culture conditions using three principal factors (nitrate limitation, salinity and light intensity), and the optimization of ultrasonic extraction in function of three factors (time, temperature, and chloroform/methanol-solvents ratio). The determination of the fatty acids profile produced from the obtained lipids can help us to quantify the biodiesel quality.

## Methods

### Conditions of *Chlorella* sp. cultivation

A *Chlorella* sp. microalga was preserved into a 1000 mL-Erlenmeyer flask containing 50 mL of inoculum and 250 mL of F/2-standard seawater medium consisting of (per liter): 1 mL of NaNO_3_ (75 g · L^− 1^), 1 mL of NaH_2_PO_4_ (5 g · L^− 1^), 1 ml of metal solution, and 0.5 mL of vitamin solution.

Cultures of *Chlorella* sp. were maintained at 25 °C and continuously illuminated at a photosynthetic light intensity of 160 μmol photons · m^− 2^ · s^− 1^ (TL5 tungsten filament lamps; Philips Co., Taipei, Taiwan) in three replicates.

For the optimization of culture conditions, the microalga (a) was grown into a 250 mL-Erlenmeyer flask containing 150 mL of culture medium composed of inoculum (10%), a modified-F/2 medium, and (b) exposed to different photosynthetic light intensities.

### Modeling of experimental kinetics

The experimental kinetics was carried out during the strain cultivation. Samples were taken every 24 or 48 h and then a direct visual cell counting was established under an optical microscope (40×) by using a Malassez cell-counting protocol. The results are presented in terms of microalgal cell number per mL in function of growth time. Three different models were chosen for cells growth kinetics prediction (Table 1): logistic, logistic-with-lag and modified-Gompertz [2, 4].

**Table 1:** Used models for cells growth kinetics prediction

Model	Expression	Parameters	Equation n°
Logistic	$X(t)=\frac{X_0\cdot {e}^{\left({\mu}_{\mathrm{max}}\cdot t\right)}}{1-\frac{X_0}{X_{\mathrm{max}}}\cdot \left(1-{e}^{\left({\mu}_{\mathrm{max}}\cdot t\right)}\right)}$	*μ*_max_; *X*_max_	(1)
Logistic- with-lag	$X(t)={X}_0+\frac{X_{\mathrm{max}}-{X}_0}{1+{e}^{\left\{\left(\frac{4\cdot {\mu}_{\mathrm{max}}}{X_{\mathrm{max}}-{X}_0}\right)\cdot \left(\lambda -t\right)+2\right\}}}$	*μ*_max_; *X*_max_; *λ*	(2)
Modified- Gompertz	$X(t)={X}_0+\left({X}_{\mathrm{max}}-{X}_0\right)\cdot {e}^{\left\{-{e}^{\left(\frac{\mu_{\mathrm{max}}\cdot {e}^1}{X_{\mathrm{max}}-{X}_0}\right)\cdot \left(\lambda -t\right)+1}\right\}}$	*μ*_max_; *X*_max_; *λ*	(3)

X and *X*_max_ refers to the actual (at time t in day) and the maximum cell number, respectively; *X*_0_ is the initial cell number at initial time 0 (X_0_ = 187 cells ∙ mL^− 1^ in this study); λ: an additional term (day)

A Matlab algorithm was carried out and applied to identify the models’ parameters using the fitting procedure consisting in the comparison the experimental data to the calculated ones. In fact, this procedure of *Chlorella* sp. growth data was established using non-linear least squares regression method. The determination coefficient (R^2^), the adjusted determination coefficient (Adj R^2^), the sum of squared errors (SSE), and the root means squared error (RMSE) were chosen in this work to quantify the models fitting quality. All models’ coefficients were determined with a 95% confidence interval (corresponding to *p* < 0.05).

### Lipids production optimization

The determination of the optimal conditions for lipids production from the *Chlorella* sp. microalga was obtained using an experimental design based on the response-surface methodology (RSM). Three factors were tested in this work: extracellular NaNO_3_ concentration (0 ≤ [NaNO_3_] ≤ 2 mL · L^− 1^), salinity of the culture medium (16 ≤ [NaCl] ≤ 32) and the applied light intensity, LI (153.2 ≤ LI ≤ 311.1 μmol · m^− 2^ · s^− 1^). Sixteen tests were performed in duplicate under different conditions following the experimental design requirement (Table 2). The experimental tests were carried out in two intervals (or two blocks) with different environmental conditions: the first eight tests were realized in the first one, and the rest in the second one. This step is very important because it tests the reproducibility of the experience.

**Table 2:** Established experiments for lipid content production and experimental response

Runs	Type	Block	[NaNO_3_] (mL · L^− 1^)	[NaCl] (−)	Light Intensity (μmol · m^− 2 ^· s^− 1^)	Lipid content (%)	
						Essay 1	Essay 2
1	Factorial points	1	0	16	153.2	11.2	6.1
2		1	2	16	153.2	8.2	6.5
3		1	0	32	153.2	12.6	12.1
4		1	2	32	153.2	8.6	14.7
5		1	0	16	311.1	15.8	15.3
6		1	2	16	311.1	7.1	7.2
7		1	0	32	311.1	12.8	10.1
8		1	2	32	311.1	6.8	4.2
9	Star points	2	0	24	163.6	4.9	5.4
10		2	2	24	163.6	16.1	9.9
11		2	1	16	163.6	18.0	18.1
12		2	1	32	163.6	19.0	17.3
13		2	1	24	153.2	17.3	18.6
14		2	1	24	311.1	14.7	14.7
15	Center points	2	1	24	163.6	15.5	19.7

### Extraction optimization

Total lipids extraction was carried out from dry biomass based on the method of Folch et al. [10]. The dry cells from 50 mL of culture were extracted using 4 mL of chloroform/methanol. In this step, the influences of three factors on lipids extraction were tested. Tested in this study were the volumetric ratio chloroform/methanol (1/1, 2/1, and 3/1 *v*/*v*); the ultrasonic exposure time (6, 18, and 30 min), and the extraction temperature (30, 45, and 60 °C). The ultrasonication was carried out with an “ultrasonic” bath (ISOLAB, Germany; tank dimensions: 150 × 138 × 65 mm^3^; tank volume: 1.3 L; ultrasonic power: 60 W; frequency: 40 kHz). The obtained mixture of each experiment was agitated for 15 min in orbital shaker at 100 rpm at room temperature. The extracts were centrifuged (2 h at 100 rpm) and the organic phase was recovered. Finally, the solvent phases were combined and evaporated to yield the lipids content, calculated within eq. (4):

4 $$ Lipids\ content\ \left(\%\right)={W}_L/{W}_A\times 100 $$

where W_L_ (g): extracted lipids weight and W_A_ (g): dry algae biomass.

Table 3 presents the adopted experimental design for the extraction process and the obtained results.

**Table 3:** Established experiments for extraction process and experimental response

Runs	Type	Time (min)	Temperature (°C)	Chloroform/Methanol (*v*/*v*)	Lipid content (%)	
					Essay 1	Essay 2
1	Factorial points	6	30	1/1	15.8	14.4
2		30	30	1/1	13.3	14.2
3		6	60	1/1	8.4	9.3
4		30	60	1/1	7.9	9.4
5		6	30	3/1	13.4	10.3
6		30	30	3/1	11.6	12.7
7		6	60	3/1	8.5	7.4
8		30	60	3/1	12.9	12.5
9	Star points	6	45	2/1	13.8	13.6
10		30	45	2/1	19.1	20.3
11		18	30	2/1	10.4	10
12		18	60	2/1	21.4	22.6
13		18	45	1/1	5.9	7.9
14		18	45	3/1	13.6	13.2
15	Center points	18	45	2/1	7.8	7.4

### Analysis of fatty acid methyl esters

Fatty acid analysis was performed after lipid extraction. The esterification of total fatty acids was carried out by a catalyst dissolved in methanol.

In the present work, the obtained lipid quantity was poured in 200 μl of hexane and 100 μl of KOH (1 N KOH in 2 N methanol) for the methylation, i.e. the sum of the interactions which take place between fatty acids and methanol. The fatty acids were analyzed by gas chromatography (GC) with electron ionization (70 eV), capillary column (length of 30 m, inner diameter of 0.25 mm and film thickness of 1 μm) and helium (1 ml  min^-1^) as carrier gas. 7 μl of the sample were injected with a dilution of 1/5 at a temperature set at 200 °C. The oven temperature was initially maintained at 50 °C for 1.5 min, then increased sequentially to 150 °C with a 15 °C · min^-1^ ramp for 8 min and finally at 200 °C (15 °C · min^-1^) for 23 min. The pressure is set at 165 kPa [11].

### Modeling and statistical study

Equation 5 presents the general form of the adopted second-degree model with interactions for the lipids production (see Lipids production optimization) and for lipid extraction (see Extraction optimization) modelling:

5 $$ {\widehat{Y}}_i={\beta}_0+\sum \limits_{i=1}^3{\beta}_i\cdot {X}_i+\sum \limits_{\begin{array}{l}i=1\\ {}i\ne j\end{array}}^3{\beta}_{ij}\cdot {X}_i\cdot {X}_j+\sum \limits_{i=1}^3{\beta}_{ii}\cdot {X}_i^2 $$

where ${\widehat{Y}}_i$: calculated lipids content (%); *β*_0_, *β*_*i*_, *β*_*ij*_, and *β*_*ii*_: constant, linear, interaction and quadratic coefficients of model, respectively; *X*_*i*_: factor level.

The coefficients *β*_0_, *β*_*i*_, *β*_*ij*_, and *β*_*ii*_ were calculated by the mean square method using the experimental matrix shown in Tables 2 and 3.

All statistical tests were performed with STATISTICA 13.0 Software, StatSoft, Inc.. The chosen confidence interval is of the order of 95%, corresponding to *p* < 0.05.

## Results

### Experimental kinetics and modelling results

**Fig. 1:**
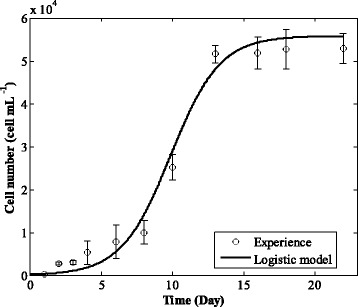
Growth kinetics of *Chlorella* sp. – Experimental data and modeling results

Figure 1 presents the experimental growth kinetics of the *Chlorella* sp. microalga. It is clear that, in 22 days, its growth presents five usual growth phases out of six: a lag phase (3 first days); an exponential phase (from day 3 to day 6); a linear phase (from the 6th to the 13th); a declining growth phase (from the 13th to the 16th); and a stationary phase (from the 16th to the 22nd) [1, 12].

Table 4 presents the fitting results of different models. In fact, all models present a high fitting quality for the experimental growth kinetics of *Chlorella* sp. with high R^2^ and Adj R^2^, and low SSE and RMSE. The most important model presenting the highest quality is the Logistic model. It is chosen in this work to fit the experimental data as depicted in Fig. 1 above.

**Table 4:** Kinetics modeling results

Model	Model’s parameters			Statistical parameters			
	*μ*_max_ (day^− 1^)	*X*_max_ (cells·mL^− 1^)	*λ* (day)	R^2^ (%)	Adj R^2^ (%)	SSE (cells · mL^−1^)^2^	RMSE (cells · mL^− 1^)
Logistic	0.5778	5.582 10^4^	–	94.36	93.79	135.8	3.685
Logistic- with-lag	9966	5.453 10^4^	7.182	93.21	91.71	163.3	4.260
Modified- Gompertz	5541	6.017 10^4^	4.250	93.34	91.86	160.3	4.220

The obtained value of *μ*_max_ corresponding to 0.0242 h^− 1^ is higher than those obtained in the case of the *Tetraselmis* sp. microalga in different photoautotrophic conditions [2, 4]. In addition, it can be considered like competitive to results obtained in the case of *Chlorella vulgaris* grown with a CO_2_ biofixation and considering the coupled effects of light intensity and dissolved inorganic carbon via photobioreactor [13].

### Optimization of lipids production

Table 2 shows the obtained experimental results for all tested culture conditions. Table 5 reports on the results of the Student test for the lipids production optimization, with the coefficients of all studied factors, their interactions and their quadratic terms.

**Table 5:** Student test results for lipids production

	Coefficient	SD of coefficient	t	*p*-value
Constant	−32.738	93.242	− 0.351	0.729
Block	−0.828	2.336	−0.354	0.727
[NaNO_3_]	21.260	5.592	3.802	0.001^b^
[NaNO_3_]^2^	−8.525	2.413	−3.533	0.002^b^
[NaCl]	0.111	1.827	0.061	0.952
[NaCl]^2^	0.008	0.038	0.207	0.838
Light intensity	0.371	0.751	0.494	0.627
Light intensity^2^	−0.001	0.002	−0.389	0.701
[NaNO_3_] × [NaCl]	0.048	0.087	0.547	0.590
[NaNO_3_] × Light intensity	−0.028	0.008	−3.453	0.003^b^
[NaCl] × Light intensity	−0.002	0.001	−2.247	0.037^a^

^a^significant effect; ^b^highly significant effect

It is clear that the nitrate concentration has a very important influence on lipids production, since both simple and quadratic terms of [NaNO_3_] are highly significant (Table 5, *p* < 0.005). In fact, the corresponding coefficients (21.260 and − 8.525, respectively) are very high compared to the other ones. The simple-effect nitrate concentration has a positive influence on lipids production, while the quadratic effect decreases it. Both interactions involving the light intensity factor, i.e. [NaNO_3_] × Light intensity (*p* < 0.005) and [NaCl] × Light intensity (*p* < 0.05) are very significant because of the importance of their coefficients compared to the corresponding standard error. Negative effects are noted for those interactions, decreasing the lipids production.

Having blocks not significant is an important result for our study, because this means that all results are reproducible (*p* > 0.05). Results are also repeatable because of the low values of standard deviation SD of experimental data (0 ≤ SD ≤ 4.38). In fact, it can be seen that all repeated experimental lipids contents are in the same order for each condition (Table 2). This explains the low values of SD and the repeatability of experimental results. The obtained model presents an acceptable quality of fitting for experimental data with an R^2^ of 78.46% and an Adj R^2^ of 67.13%.

**Fig. 2:**
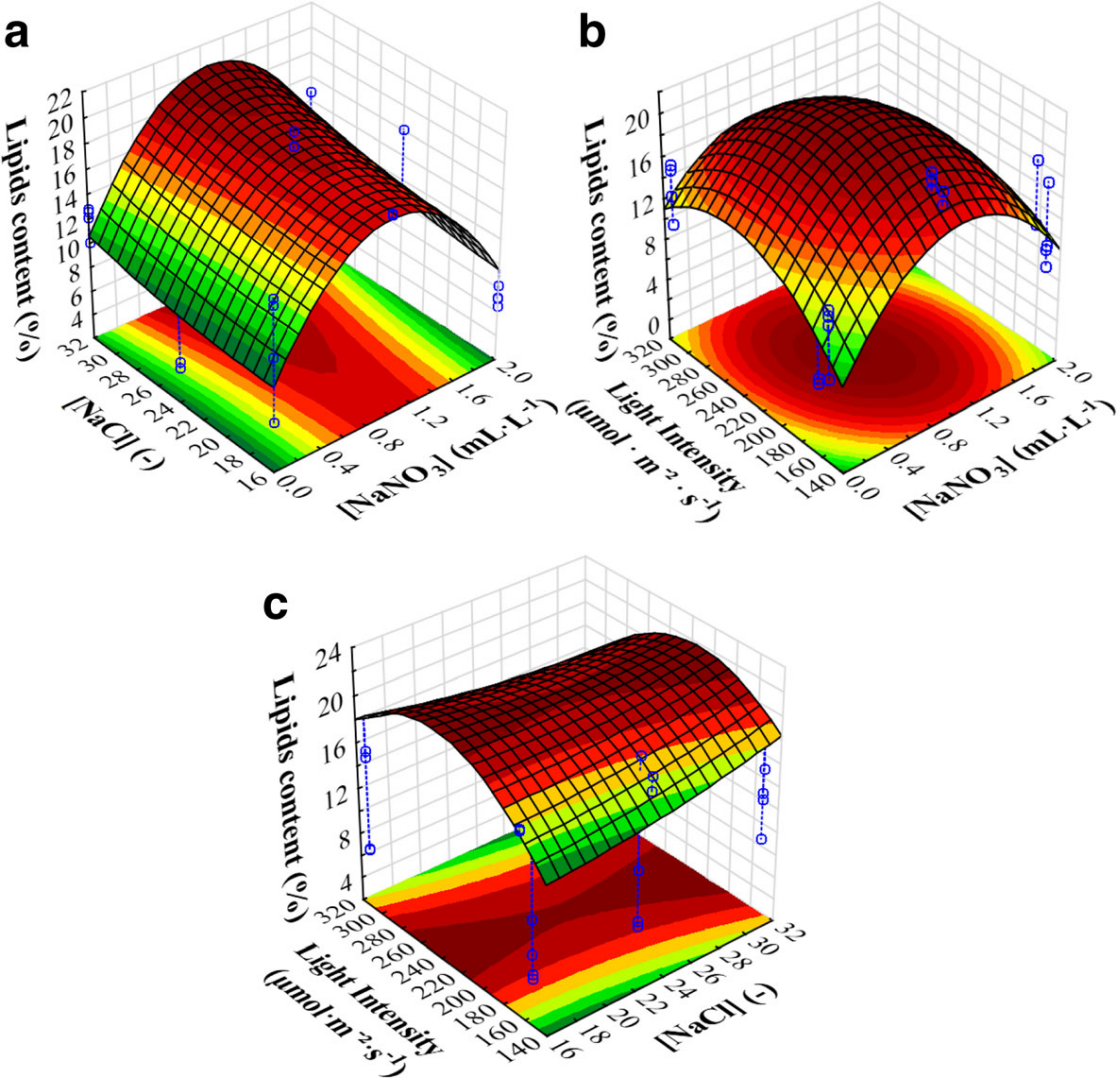
2D and 3D representations of lipids contents in function of (**a**): [NaNO_3_] and [NaCl] (at Light intensity = 163.6 μmol · m^− 2^ · s^− 1^); (**b**): [NaNO_3_] and light intensity (at [NaCl] = 24); and (**c**): light intensity and [NaCl] (at [NaNO_3_] = 1 mL · L^− 1^)

Figure 2 presents the 2D and 3D representations of the lipids content in function of different studied factors. It can be seen that the lipids production is maximal when using the median (1 mL · L^− 1^) of the tested range of [NaNO_3_] (Fig. 2a and b) and using the high intensity of light (Fig. 2a and c). Results equally show that there is no impact of NaCl concentration (Fig. 2b and c). In brief, we can affirm that the previously obtained results are confirmed graphically.

**Fig. 3:**
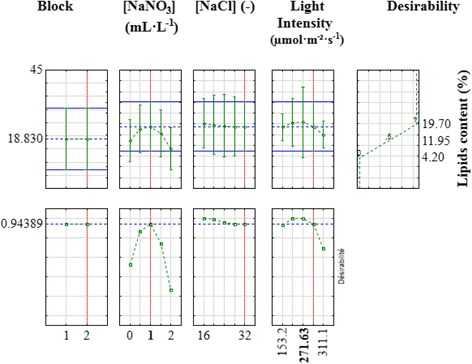
Determination of the maximum for lipids production in function of culture conditions

The determination of the lipids production maximum (18.83%) was carried out via the variation of all factors simultaneously (Fig. 3). This result was obtained with a desirability of 94.4%. In fact, the maximum of lipids production is obtained at lower concentration of NaNO_3_ (1 mL · L^− 1^) compared to the standard culture conditions used for biomass culture, at non-diluted sea water knowing by salinity of 32, and at high light intensity of 271.63 μmol · m^− 2^ · s^− 1^. These results are in accordance with prior findings since the limitation of extracellular nitrate concentration and the increase of applied light intensity induce, for different microalgae, lipids accumulation inside the microalgal cells [14–17].

### Optimization of lipids extraction

**Fig. 4:**
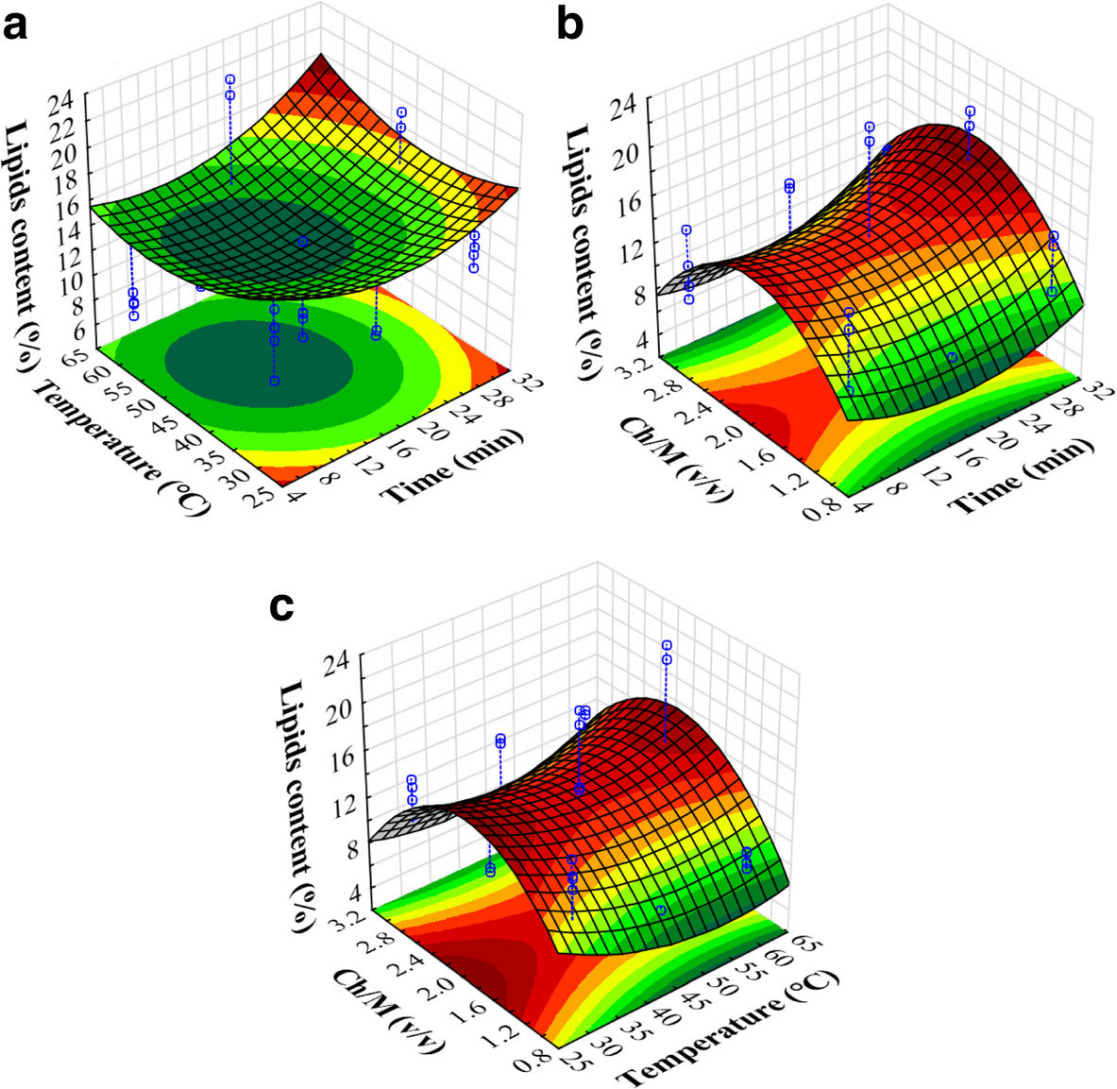
2D and 3D representations of extracted lipids content in function of: (**a**) time and temperature (at Chlorform/Methanol = 2/1 *v*/*v*); (**b**) time and Chloroform/Methanol (at temperature = 45 °C); and (**c**) temperature and Chloroform/Methanol (at time = 18 min)

The best condition yielding the best lipids content was then chosen to be used for the extraction optimization. The obtained experimental values of extracted lipids range from 5.9 to 22.6% (Table 3 and Fig. 4). These values are shown to be in the same order as those obtained for the *Chlorella vulgaris* (albeit using different extraction techniques) found to be between 7.6 and 32.0% [8] or 10.58 to 22.59% for the [18]).

In this work, we have demonstrated that all results are repeatable since the standard deviation is from 0.14 to 2.19%. It can also be seen that tests 10 (Y = 19.7 ± 0.8%) and 12 (Y = 22 ± 0.8%) present higher lipids percentages. In addition, they give a very highly statistically significant difference compared to corner points (*p* < 0.001) and to center points (*p* < 0.003) of the experimental matrix. It is crucial to note that runs 10 and 12 are established in the central level of the chloform/methanol factor (Ch/M) (2/1). Table 6 presents the result of the Student test. It is clear that only the quadratic term of the solvents ratio (Ch/M)^2^ has a significant effect on the extraction of lipids (*p* < 0.05). This result shows the importance of the solvents composition in the extraction of lipids. The same findings have been reported in previous studies [8, 19]. Time and temperature with linear and quadratic terms and all interactions do not have significant effects on response.

**Table 6:** Student test results for lipids extraction

Factors	Coefficient	SD of coefficient	t	*p*-value
Constant	19.721	16.951	1.163	0.258
Time	− 0.689	0.573	−1.203	0.243
Time^2^	0.013	0.013	0.972	0.343
Temperature	−0.712	0.773	−0.920	0.369
Temperature^2^	0.005	0.008	0.652	0.522
Ch/M	15.154	8.360	1.813	0.085
(Ch/M)^2^	−4.728	1.875	−2.521	0.020^a^
Time × Temperature	0.004	0.006	0.658	0.518
Time × Ch/M	0.069	0.089	0.776	0.447
Temperature × Ch/M	0.067	0.071	0.941	0.358

^a^significant effect

**Fig. 5:**
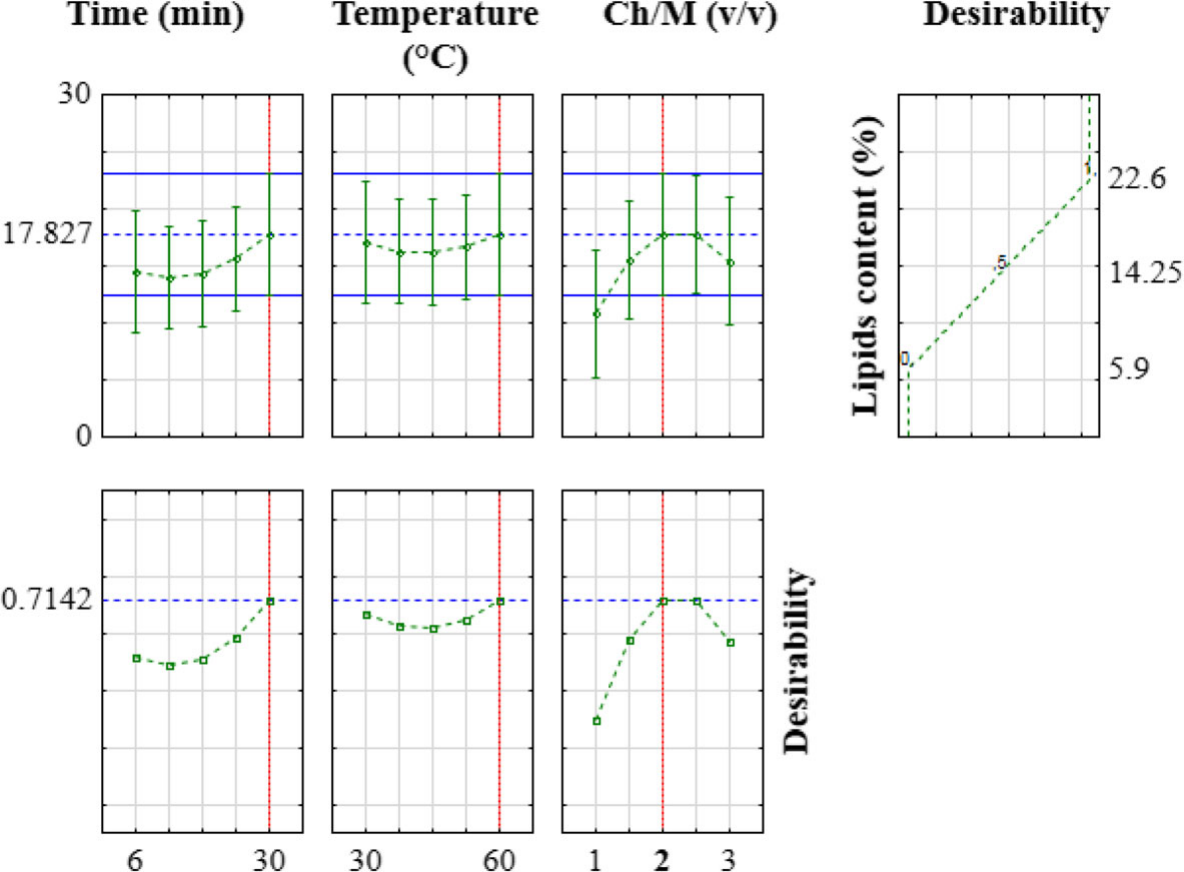
Optimal conditions for a maximum lipids extraction from *Chlorella* sp.

Figure 4 shows the 2D and 3D presentations of the extracted lipids in function of various factors. Figure 4a depicts lipids extraction evolution in function of temperature and the time of ultrasound exposition (at central level of Ch/M factor). In this case, the response presents an elliptical form and a minimum response. Figure 4b reveals the lipids extraction’s dependence on time and chloroform/methanol solvents ratio. In fact, the form in this domain is hyperbolic and it presents a fitted maximum at high ultrasonic time and for a central value of Ch/M. Figure 4c points out a hyperbolic form of lipids extraction in function of two factors: Ch/M and temperature. In this case, the maximum response is shown in high temperature and in central level of Ch/M. The optimal condition to obtain maximum lipid extraction was described in Fig. 5. The obtained result shows that the required chloroform/methanol ratio must be equal to 2/1; time and temperature must be in high levels, corresponding to 30 min and 60 °C, respectively. As a case in point, Park et al. (2014) demonstrate that a chloroform/methanol ratio of 2/1 is the best for lipids extraction from *Chlorella vulgaris* microalgae. The optimal condition we determine gives a calculated maximum of 17.83% and can attain a value of 22.60%, a value very close to those published in the literature on the topic [8, 9, 19].

### Fatty acids profiles proprieties

The produced lipids in optimal conditions were analyzed. Fatty acids profiles showed the presence of seven fatty acid methyl esters: 0.43% of lauric acid (C12:0), 1.22% of myristic acid (C14:0), 51.69% of palmitic acid (C16:0), 20.55% of oleic acid (C18:1), 7.84% of linoleic acid (C18:2), 7.78% of linolenic acid (C18:3), and 10.5% of arachidic acid (C20:0).

In fact, it is clear that the fatty acid methyl esters derived from the *Chlorella* sp. microalga cultured at the determined optimal conditions are characterized by a high amount of saturated and monounsaturated chain fatty acids of ≈72% of total fatty acids (principally C16:0 and C18:1) and a low-level amount of polyunsaturated chain fatty acids of ≈15% of total fatty acids (C18:2 and C18:3). This is very suitable for high-quality biodiesel production [5, 15, 20, 21]. In fact, biodiesel oxidation stability is always affected by the high level of polyunsaturated fatty acids, that tend to oxidize rapidly. This impacts the storage stability negatively, which is critical for fuel applications [5, 20].

The obtained results are very similar to those of previous works showing different stress conditions applied during culture of different microalgae [5, 15, 20, 21].

## Conclusion

Three principal axes were treated in this work. First of all, the experimental culture kinetics of *Chlorella* sp. were established in standard medium. The modelling of the experimental data was also carried out via three models: logistic, logistic-with-lag and modified Gompertz. The most important model generating the best fitting quality is the logistic model. The second part of the present work was interested in the optimization of lipids production from *Chlorella* sp. via response-surface methodology. The better condition given the maximum lipids production is 1 mL · L^− 1^ of [NaNO_3_], 32 of salinity, and high light intensity of 271.63 μmol · m^− 2^ · s^− 1^. The corresponding fatty acid profile makes *Chlorella* sp. microalga a viable alternative to traditional sources of fossil fuel and an important feedstock source of high-quality biofuel. The third part is about the optimization of ultrasonic extraction of lipids from *Chlorella* sp. microalgal cells. The studied factors were the ultrasonic extraction period, the temperature and the chloroform-methanol solvents ratio. The best extraction can be established with longer time (30 min), higher temperature (60 °C) and with a chloroform-methanol solvents ratio of 2/1.

## Abbreviation

Adj R^2^: Adjusted determination coefficient (dimensionless)
*p*: Value of probability (dimensionless)
R^2^: Determination coefficient (dimensionless)
RMSE: Root Means Squared Error
SD: Standard Deviation
SSE: Sum of Squared Errors
t: Student coefficient (dimensionless)
X: cell number per mL
μ_max_: Maximum specific growth rate (day^− 1^)

## References

[1] Lee E, Jalalizadeh M, Zhang Q (2015). Growth kinetic models for microalgae cultivation: a review. Algal Res.

[2] Dammak M, Hadrich B, Barkallah M, Hentati F, Hlima HB, Pichon C, Denis M, Fendri I, Michaud P, Abdelkafi S (2018). Modelling *Tetraselmis* sp. growth-kinetics and optimizing bioactive-compound production through environmental conditions. Bioresour Technol.

[3] Dammak M, Hadrich B, Miladi R, Barkallah M, Hentati F, Hachicha R, Laroche C, Michaud P, Fendri I, Abdelkafi S (2017). Effects of nutritional conditions on growth and biochemical composition of *Tetraselmis* sp. Lipids Health Dis.

[4] Mohamed MS, Tan JS, Kadkhodaei S, Mohamad R, Mokhtar MN, Ariff AB (2014). Kinetics and modeling of microalga *Tetraselmis* sp. FTC 209 growth with respect to its adaptation toward different trophic conditions. Biochem Eng J.

[5] Arorra N, Patel A, Pruthi PA, Pruthi V (2016). Synergistic dynamics of nitrogen and phosphorous influences lipid productivityin *Chlorella minutissima* for biodiesel production. Bioresour Technol.

[6] Dammak M, Mareike Haase S, Miladi R, Ben Amor F, Barkallah M, Gosset D, Pichon C, Huchzermeyer B, Fendri I, Denis M, Abdelkafi S (2016). Enhanced lipid and biomass production by a newly isolated and identified marine microalga. Lipids Health Dis.

[7] Liu J, Yuan C, Hu G, Li F (2012). Effects of light intensity on the growth and lipid accumulation of microalga *Scenedesmus* sp. 11-1 under nitrogen limitation. Appl Biochem Biotechnol.

[8] Garoma T, Janda D (2016). Investigation of the effects of microalgal cell concentration and electroporation, microwave and ultrasonication on lipid extraction efficiency. Renew Energy.

[9] Park J-Y, Oh Y-K, Lee J-S, Lee K, Jeong M-J, Choi S-A. Acid-catalyzed hot-water extraction of lipids from Chlorella vulgaris. J Bioresour Technol. 2014;153:408–12. Short Communication. 10.1016/j.biortech.2013.12.065 24393546

[10] Folch J, Lees M, Sloane Stanley GH (1957). A simple method for the isolation and purification of total lipids from animal tissues. J Biol Chem.

[11] Patel A, Sindhu DK, Arora N, Singh RP, Pruthi V, Pruthi PA (2015). Biodiesel production from non-edible lignocellulosic biomass of *Cassia fistula* L. fruit pulp using oleaginous yeast *Rhodosporidium kratochvilovae* HIMPA1. Bioresour Technol.

[12] Andersen RA (2005). Algal culturing techniques.

[13] Chang HX, Huang Y, Fu Q, Liao Q, Zhu X. Kinetic characteristics and modeling of microalgae *Chlorella vulgaris* growth and CO_2_ biofixation considering the coupled effects of light intensity and dissolved inorganic carbon. Bioresour Technol. 2016;206:231-8. 10.1016/j.biortech.2016.01.087 26866758

[14] Chen M, Tanga H, Maa H, Hollandb TC, Nga KYS, Salley SO (2011). Effect of nutrients on growth and lipid accumulation in the green algae *Dunaliella tertiolecta*. Bioresour Technol.

[15] Damiani MC, Popovich C, Constenla D, Martínez A, Doria E, Longoni P, Cella R, Nielsen E, Leonardi P (2014). Triacylglycerol content, productivity and fatty acid profile in PVUW12. J Appl Phycol.

[16] Feng P, Deng Z, Fan L, Hu Z (2012). Lipid accumulation and growth characteristics of *Chlorella zofingiensis* under different nitrate and phosphate concentrations. J Biosci Bioeng.

[17] Pal D, Goldberg IK, Cohen Z, Boussiba S (2011). The effect of light, salinity and nitrogen availability on lipid production by *Nannochloropsis* sp. Appl Microbiol Biotechnol.

[18] Bhattacharya S, Maurya R, Mishra SK, Ghosh T, Patidar SK, Paliwal C, Chokshi K, Pancha I, Maiti S, Mishra S (2016). Solar driven mass cultivation and the extraction of lipids from *Chlorella variabilis*: a case study. Algal Res.

[19] Mathimani T, Uma L, Prabaharan D (2017). Optimization of direct solvent lipid extraction kinetics on marine trebouxiophycean alga by central composite design–bioenergy perspective. Energy Convers Manag..

[20] Anahas AMP, Muralitharan G (2018). Characterization of heterocystous cyanobacterial strains for biodiesel production based on fatty acid content analysis and hydrocarbon production. Energy Convers Manag.

[21] Xu H, Miao X, Wu Q (2006). High quality biodiesel production from a microalga *Chlorella protothecoides* by heterotrophic growth in fermenters. J Biotechnol.

